# 

**Published:** 1867-06

**Authors:** 


### CONTENTS.

### ORIGINAL CONTRIBUTIONS.

ART. XXIII. CASE OF HYDROPHOBIA. By DANIEL T. NELSON, Prof. of Physiology and Histology ........ 321

ART. XXIV. NOTES ON SPANISH SAFFRON. By HENRY BIROTH, Chicago. (Read to the Chicago College of Pharmacy.) ........ 325

ART. XXV. RECOVERY FROM BACKWARD CURVATURE OF THE SPINE, WITH REMOVAL OF THE DEFORMITY. By JULIEN S. SHERMAN, M.D., Chicago. ........ 328

ART. XXVI. REPORT ON THE SANITARY CONDITION OF THE CITY, ETC. By N. S. DAVIS, M.D., Member of Sanitary Committee. (Read to the Chicago Medical Society, April 27th, 1867.) ........ 329

### PROCEEDINGS OF SOCIETIES.

American Medical Association. Eighteenth Annual Session ........ 331
- Address of Greeting ........ 332
- Meetings of the Sections ........ 332
- Entertainments and Receptions. ........ 333
- Private Receptions. ........ 333
- The President's Address. ........ 333
- Report on Rank of Medical Men in the Navy ........ 335
- Prize Essays.—Reform in Medical Colleges ........ 340
- Prize Essay Matters.—Papers Presented. ........ 341
- Medical Statistics in the Army. ........ 342
- Medical Literature.—Entertainment at Hon. Geo. H. Pendleton's ........ 343
- Female Physicians ........ 343
- Report on Insanity.—Nominations and Places of Meeting. ........ 344
- Steamboat Excursion. ........ 347
- Place of Next Meeting.—The Cinchona Tree ........ 348
- Papers on Cholera.—Further Nominations. ........ 349
- Amendments to the Constitution.—Compensation to the Secretary. ........ 349
- Resolutions of Thanks ........ 349
- Unofficinal Medicines. ........ 350
- Female Physicians ........ 351
- The Chronic Insane ........ 352

Convention of Teachers of Medical Colleges in the United States ........ 353

Morgan County Medical Society ........ 363

### SELECTIONS.

On the Influence of Hypodermic Injection upon the Science of Toxicology. By S. P. Duffield, Ph. D. ........ 373

Death from Chloroform at Bellevue Hospital ........ 378

The New Treatment of Rheumatism ........ 379

### EDITORIAL.

Personal. ........ 380

Bellevue Place. ........ 380

Money Receipts from April 22d to May 27th ........ 380

Mortality Report for the Month of April. ........ 381

